# Construction of nursing-sensitive quality indicators for hemodialysis vascular access: a Delphi study

**DOI:** 10.3389/fpubh.2026.1858484

**Published:** 2026-07-13

**Authors:** Yingjun Zhang, Li He, Sikai Tang, Yueshan Gao, Hui Chen, Mei Shi, Lin Chen

**Affiliations:** Hemodialysis Center, Department of Nephrology, West China Hospital, Sichuan University/West China School of Nursing, Sichuan University, Chengdu, China

**Keywords:** Dephi method, hemodialysis, nursing quality, nursing-sensitive quality indicator, vascular access

## Abstract

**Aims:**

The aim of this study was to develop a set of quality indicators for hemodialysis vascular access nursing.

**Design:**

A two-round Delphi study was conducted from August to September 2025.

**Methods:**

Based on the Donabedian structure–process–outcome theory model, this study retrieved, evaluated, and synthesized the best evidence on quality indicators for hemodialysis vascular access nursing. A questionnaire was developed through evidence-based analysis and semistructured interviews. An indicator system was established using the Delphi method, and an analytic hierarchy process (AHP) was employed to determine the weights of each indicator. A total of 16 experts from 14 hospitals across nine provinces in China completed two rounds of Delphi surveys.

**Results:**

After two rounds of consultation, the experts reached a consensus on the definitions of the indicators, calculation formulas, and data collection methods. They established a quality indicator system for hemodialysis vascular access nursing, which includes 3 primary indicators and 15 secondary indicators.

**Conclusion:**

The developed nursing quality-sensitive indicator system for hemodialysis vascular access was constructed through a rigorous Delphi process and aligns with the practical features of hemodialysis vascular access nursing management. It may serve as a reference basis for evaluating the quality of vascular access nursing, pending further clinical validation.

**Implications for the profession and patient care:**

This framework offers a structured approach to monitoring vascular access nursing quality. Its potential to reduce complications and improve patient outcomes requires confirmation through future clinical implementation studies.

**Impact:**

The indicators identified in this study are highly consistent with those in clinical practice, demonstrating sensitivity and practicality. The hemodialysis centers of relevant medical institutions may consider using this quality indicator system for routine data collection. Its utility for quality improvement should be further evaluated in clinical practice.

**Reporting method:**

This study was reported in line with the Conducting and Reporting of Delphi studies (CREDE) guidance on Delphi studies.

## 1 Introduction

Chronic kidney disease (CKD) has become a global public health concern. The latest data from The Lancet (1) predict that in 2023, approximately 788 million adults worldwide will suffer from CKD, with a prevalence as high as 14.2%. Some CKD patients may progress to end-stage renal disease (ESRD). The main treatment methods for ESRD patients include hemodialysis (HD), peritoneal dialysis (PD), and kidney transplantation. The vast majority of patients choose HD as their main treatment method, and the global number of patients on dialysis, which has continuously increased over the past 30 years, is predicted to reach 3.57 million in 2023 (2). According to data from the China Kidney Disease Network (CK-NET), the number of hemodialysis patients in China exceeded one million at the end of 2024.

Vascular access serves as the “lifeline” for patients receiving maintenance hemodialysis (MHD). Common vascular access methods include autologous arteriovenous fistulas (AVFs), arteriovenous graft fistulas (AVGs), and central venous catheters (CVCs). The functional status of vascular access is closely associated with patient mortality and quality of life. Therefore, maintaining the normal function of vascular access is a shared responsibility between health care providers and patients, with hemodialysis nurses playing a leading role in this process. Hemodialysis nurses, who face a high volume of daily tasks such as AVF punctures and CVC care, are the most frequent users of vascular access for MHD patients. Increasing evidence indicates that nursing techniques significantly influence the lifespan of vascular access (3–5). Relevant guidelines, both domestically and internationally, emphasize the critical role of nurses in vascular access management (6, 7), suggesting that hemodialysis vascular access nurses should take responsibility for vascular access planning, functional monitoring, the coordination of access-related procedures, and the documentation of access-related data. These measures aim to increase the quality of vascular access care and reduce the incidence of access-related complications (8, 9). Consequently, effective evaluation of nursing quality in the management of hemodialysis vascular access is essential.

## 2 Background

Nursing quality evaluation is a core component of hospital management. Nursing quality-sensitive indicators, as objective quantitative measures of clinical nursing quality, serve both as a quality assessment tool and a critical management instrument. Their results are directly associated with patient health and safety, and can objectively and authentically reflect the actual level of nursing quality, thus providing evidence to guide the improvement of nursing practice (10, 11). Nursing quality-sensitive indicators originated in the United States, first proposed by the American Nurses Association (ANA), which defined them as quantitative tools used to evaluate the process of nursing activities and their impact on patient outcomes (12). The structure–process–outcome model proposed by Donabedian (13) provides the primary framework for developing nursing quality indicators. This model identifies structural indicators as environmental factors that directly or indirectly influence nurses' patient care, encompassing material resources, human resources, and organizational structures (14). Process indicators primarily reflect specific medical service activities, representing the procedures through which patients receive care or nurses implement interventions (15). Outcome indicators measure the results of patients receiving nursing services, including service quality and clinical outcomes (16).

In recent years, exploratory studies on quality indicators in specialized and disease-specific nursing care have been gradually conducted (17–19), but research on sensitive indicators for hemodialysis vascular access nursing remains limited. Existing studies focus primarily on the construction of general hemodialysis nursing quality indicators, with only a few items related to vascular access, and the constructed indicators do not fully encompass the three dimensions of structure–process–outcome. Among the 26 hemodialysis nursing quality indicators developed by McIntyre et al. (20), only 5 items are related to vascular access, and the 11 indicators identified by Gao et al. (21) include only 3 items related to vascular access, without dimension-specific categorization. Therefore, it is important to construct scientific, effective, and hemodialysis vascular access-specific nursing quality indicators. In this study, on the basis of evidence-based principles and the Donabedian structure–process–outcome framework, the Delphi method was employed to develop hemodialysis vascular access-specific nursing quality indicators, with the goal of providing a scientific basis for the evaluation and continuous improvement of the quality of hemodialysis vascular access nursing.

## 3 The study

### 3.1 Aim

This study aimed to develop a set of quality indicators for hemodialysis vascular access nursing applicable to China based on Donabedian theory.

### 3.2 Design

This study retrieved, evaluated, and synthesized the best available evidence on quality indicators for hemodialysis vascular access nursing from domestic and international sources. On the basis of evidence-based analysis and semistructured interviews, a questionnaire was developed. Sixteen experts were subsequently consulted between August and September 2025. Through two rounds of Delphi surveys, quality indicators for hemodialysis vascular access nursing were established, and an analytic hierarchy process (AHP) was employed to determine the weights of each indicator. This study was reported in line with the Conducting and Reporting of Delphi studies (CREDE) guidance, as shown in Supplementary Table S1.

## 4 Methods

### 4.1 Creating a research team

The project team consisted of six members, including one head nurse (bachelor's degree, ≥20 years of hemodialysis experience), one chief physician and one attending physician specializing in vascular access (PhD degree, ≥10 years of vascular access experience), and three postgraduate researchers (≥8 years of hemodialysis experience). The research team was primarily responsible for developing literature search strategies, formulating interview outlines, organizing and analyzing expert opinions, and ultimately establishing sensitive indicators for hemodialysis vascular access nursing quality.

### 4.2 Literature review

A literature search was conducted in the PubMed, Cochrane Library, CINAHL, Embase, Web of Science, CNKI, Wanfang, and CMGN databases, as well as the NICE guidelines. The Chinese search terms used were hemodialysis + arteriovenous fistula/central venous catheters/vascular access + nursing quality/quality indicators/nursing quality assessment/nursing sensitive indicators, whereas the English search terms used were hemodialysis + arteriovenous fistula/central venous catheters/vascular access + nursing quality/quality indicators/nursing quality assessment/nursing sensitive indicators. The search time frame ranged from database establishment to June 30, 2025, with language restrictions set to Chinese and English, and the snowball method was also used to search the references of the included literature. The inclusion criteria were as follows: vascular access for hemodialysis; and clinical guidelines, expert consensus, evidence summaries, systematic reviews, and original studies. The exclusion criteria were as follows: studies with a C level quality assessment; studies with repeated publications of viewpoints or main content; and studies whose full text could not be obtained. The complete Boolean search strings for each database are provided in Supplementary Table S2.

**Figure 1:**
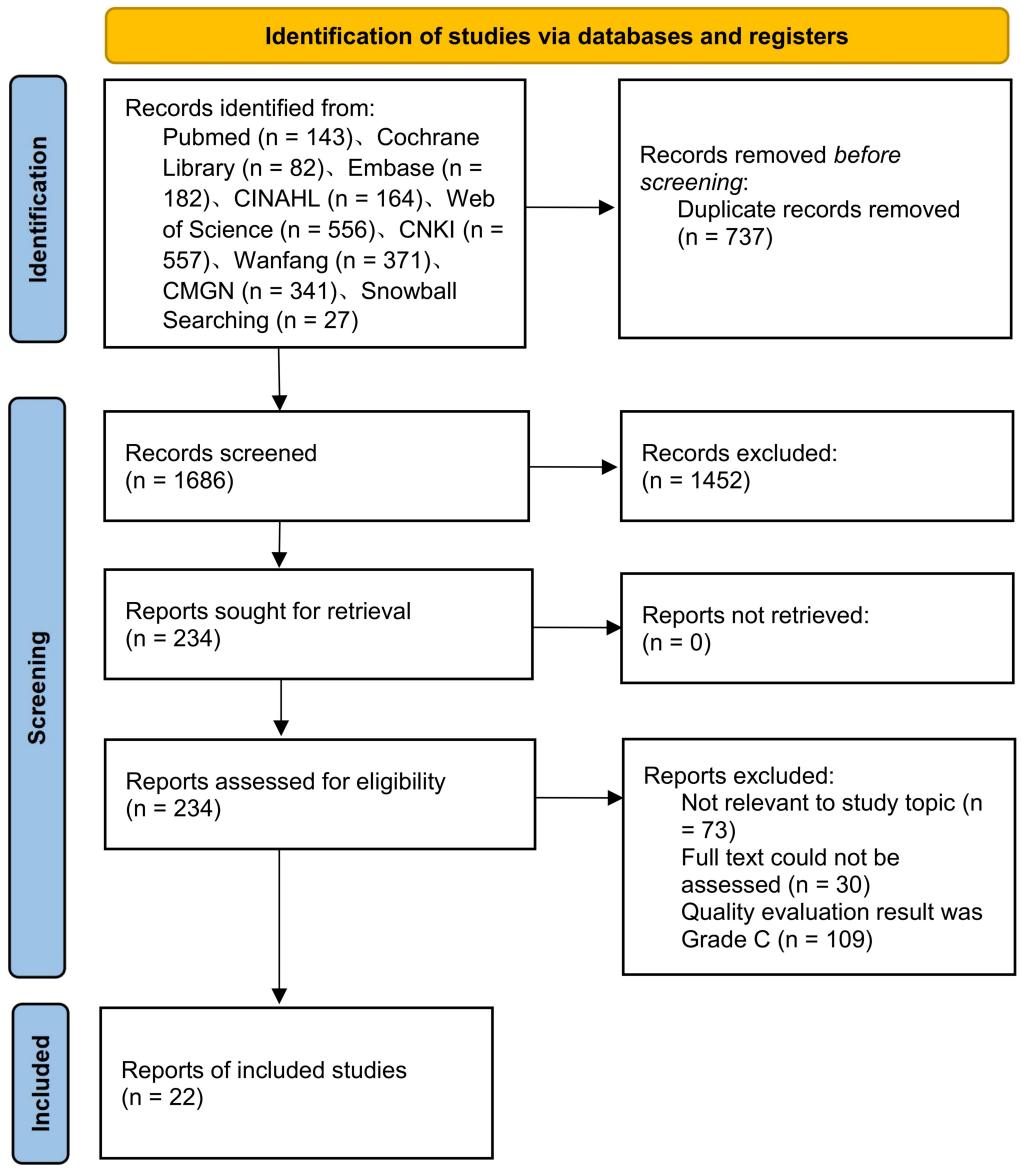
Flow diagram of the literature search and screening process. CNKI, China National Knowledge Infrastructure; CMGN, Chinese Medlive Guideline Network.

Two research team members independently screened the titles and abstracts of all retrieved records, followed by full-text assessment against the pre-specified eligibility criteria. Disagreements were resolved through discussion or by consulting a third team member. A total of 2,423 articles were retrieved. The detailed screening process, including reasons for exclusion at each stage, is presented in the PRISMA 2020 flow diagram (Figure 1). Evidence evaluation was conducted according to the evidence assessment method in the Johns Hopkins Nursing Evidence-based Practice (JHNEBP) model (22). Each eligible article was independently evaluated by two study team members. In cases of disagreement, a third member was involved in the evaluation. If a consensus could not be reached, the study team conducted a collective discussion. The quality evaluation results of the included articles were as follows: Ia, 1 article; IIIa, 2 articles; IVa, 3 articles; Ib, 5 articles; IIb, 6 articles; IIIb, 3 articles; and Vb, 2 articles. A summary of the 22 included studies and their evidence sources is presented in Supplementary Table S3. The study team organized and explained the meaning of the indicators, calculation formulas, and data collection methods.

### 4.3 Semi-structured interviews

A purposive sampling method was employed to conduct semistructured interviews with six experts (two nursing managers, two hemodialysis vascular access nurses, and two vascular access physicians). The interviews were conducted by a research team member trained in qualitative methodology. The inclusion criteria for clinical nursing experts were (1) ≥10 years of clinical nursing experience in hemodialysis; (2) intermediate professional title or above; (3) familiarity with nursing quality indicators; and (4) voluntary participation in the study. The inclusion criteria for clinical medicine experts were (1) ≥10 years of work experience in hemodialysis vascular access; (2) a senior professional title or above; and (3) familiarity with the quality management and evaluation of hemodialysis vascular access and (4) willingness to participate in the interview.

The interview outline was as follows: What do you consider to be the key points and challenges in the nursing process of hemodialysis vascular access? From your perspective, what aspects should be focused on at the core of a quality indicator system for hemodialysis vascular access nursing? Do you think the indicators extracted from the literature are applicable to the quality management of hemodialysis-related vascular access nursing? Which do you suggest adding or removing?

The interviews were conducted after obtaining informed consent from each participant, lasted 30 to 60 min, and were audio-recorded with the participants' permission. Each interview was transcribed verbatim, and the transcripts were reviewed by the interviewer to confirm accuracy. Two research team members independently coded the transcripts using thematic analysis. Any disagreements were resolved through discussion, with a third member consulted when needed. The Colaizzi 7-step analysis method was used to analyze the interview content.

Finally, corresponding indicators were extracted from documents such as the “Practical Handbook of Nursing-Sensitive Quality Indicators (2016 Edition)” compiled by the Nursing Center of the National Health and Family Planning Commission's Hospital Management Research Institute and the “Medical Quality Control Indicators for Nephrology (2020 Edition)” issued by the National Health Commission. On the basis of the literature review and interview results and using the Donabedian three-dimensional theoretical model as a framework, a preliminary set of sensitive indicators for hemodialysis vascular access nursing was established, comprising 3 structural indicators, 7 process indicators, and 6 outcome indicators.

### 4.4 Developing an expert correspondence questionnaire

The expert inquiry questionnaire comprised three sections. The first section was the introductory statement, which primarily informed the experts of the purpose of the inquiry, the definitions of the terms involved in the questionnaire, and the precautions for completing the questionnaire. The second section was the expert opinion form on sensitive quality indicators for hemodialysis vascular access nursing care. Each indicator was rated on a 5-point Likert scale, where 5 = very important, 4 = important, 3 = moderately important, 2 = not important, and 1 = very unimportant. The experts evaluated the indicators on the basis of their professional knowledge and prior experience and provided modification suggestions, including deletions or additions of relevant indicators. The third section included the experts' basic information, such as age, gender, position, professional title, main research direction, and familiarity with the inquiry content and the criteria for judging the indicators.

### 4.5 Selection of correspondence experts

In accordance with the requirements of the Delphi expert consultation method and the research objectives, this study selected 16 experts from 9 provinces and municipalities, including Sichuan, Yunnan, Beijing, Shanghai, Shandong, Chongqing, and Fujian, and others, as respondents for the letter inquiry. The inclusion criteria for the experts were as follows: had ≥10 years of dialysis-related work experience and were familiar with recent advancements in hemodialysis vascular access; had a bachelor's degree or above; had a professional title of intermediate or above; and participated voluntarily with timely completion of the expert letter inquiry. Throughout the consultation, the identities and affiliations of all panel members were kept anonymous to the other experts.

### 4.6 Establishing indicator screening criteria

Prior to expert consultation, the research team established the following criteria for indicator screening (23, 24): (1) indicators with a mean importance score >3.5, a coefficient of variation (CV) < 0.25, and a full-score rate >20% were retained; (2) indicators with a mean importance score >3.5 and a CV ≥0.25 that were deemed important on the basis of clinical experience were modified and advanced to the next round of inquiry; and (3) indicators with a mean importance score ≤ 3.5 were excluded. Qualitative disagreements on indicator wording or definitions were resolved through team discussion and majority vote.

### 4.7 Two rounds of expert consultations

**Figure 2:**
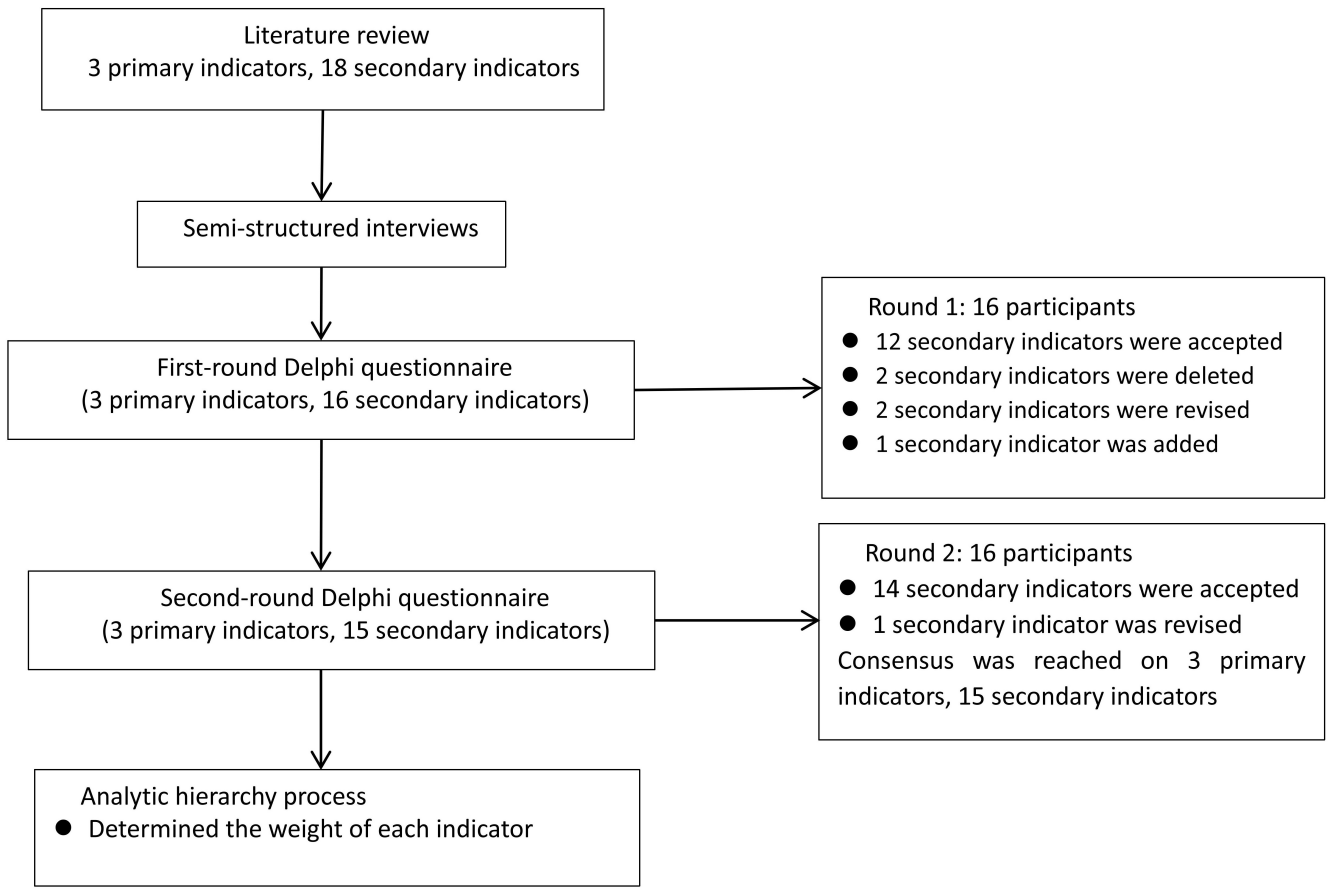
Flow chart of the two-round Delphi consultation process.

From August to September 2025, the study conducted two rounds of expert consultations. In each round, the researchers distributed the consultation questionnaires to the experts via onsite visits, WeChat, or email. The experts evaluated the indicators on the basis of their importance and the feasibility of the data collection methods, with any modifications noted in the revision column. After the first round, the research team refined the indicators using expert feedback and group discussions, generating a second-round consultation questionnaire. In addition, each expert received statistical feedback and anonymized summaries of qualitative comments from other panel members. The consultation was terminated after two rounds because all indicators met the pre-specified retention criteria and no further substantive modifications were proposed by any expert in the second round. To assess consensus stability, we compared the mean importance scores of all retained indicators between the two rounds, the mean-score change rate was less than 5%, and the coefficient of variation for each indicator decreased to below 0.25 in the second round, indicating that expert opinions had stabilized. The questionnaires were collected 2 weeks after each round, with a three-week interval between rounds (the consultation process is shown in Figure 2).

### 4.8 Analytic hierarchy process

The analytic hierarchy process (AHP) was used to determine the weight of each indicator. All calculations were performed using R 4.5.2, employing the matrix operation functions in the *stats* package and the *ahp* package for analysis. Pairwise comparisons were performed using Saaty's 1–9 scale to construct judgment matrices for each level. The maximum eigenvalue (λ_max), eigenvectors, and consistency index were calculated for each matrix. Individual initial weights were derived from the normalized eigenvectors. The consistency ratio (CR) was used to test the consistency of each judgment matrix, and a CR < 0.1 was considered acceptable. The λ_max and CR values for each judgment matrix were reported.

### 4.9 Statistical analysis

Data were entered and analyzed using Excel 2016 and SPSS 26.0 statistical software. Normally distributed measurement data are expressed as the mean ± standard deviation (Mean ± SD), whereas categorical data are presented as the frequency and percentage (%). Expert engagement was measured by the questionnaire response rate (%), with the familiarity and judgement criteria denoted as Cs and Ca, respectively. Expert authority was assessed using the authority coefficient (Cr), which was calculated as the average of Cs and Ca for the consulted experts: Cr = (Cs + Ca)/2. The coefficient of variation (CV) reflected the concentration of expert weight assignments for each indicator, with a CV < 0.25 indicating balanced scoring across indicators. Expert opinion consistency was evaluated using Kendall's W, a score ranging from 0 to 1, where higher values indicate better alignment. The significance level was set at α = 0.05.

### 4.10 Ethics

All the participants provided informed consent and voluntarily participated in this study. The study was approved by the Ethics Committee of West China Hospital, Sichuan University with approval number 2024(552). All methods were performed in accordance with the relevant guidelines and regulations.

## 5 Results

### 5.1 General expert information

In the first round, 16 questionnaires were distributed, with all 16 completed questionnaires recovered, resulting in a 100% valid recovery rate. In the second round, 16 questionnaires were distributed, with all 16 completed questionnaires recovered, which also resulted in a 100% valid recovery rate, indicating the high level of engagement among the experts in the study. Among the 16 experts who completed both rounds of correspondence, 5 were males and 11 were females; their average age was 41.74 ± 5.86 years, and their average years of work experience was 21.38 ± 7.50 years. The experts included 8 with intermediate professional titles, 8 with senior professional titles, 5 with master's degrees, and 11 with bachelor's degrees. A detailed breakdown of each expert's discipline, role, institution type, province, years of vascular-access experience, Delphi experience, and professional category is provided in Supplementary Table S4.

### 5.2 Expert authority and degree of consensus

In this study, the two rounds of expert inquiries involved the same panel of experts, with Cs = 0.894, Ca = 0.913, and Cr = 0.904. A value of Cr > 0.9 indicates a high level of expert authority. The Kendall's W values for indicator importance, calculation formulas, and data collection feasibility ranged from 0.136 to 0.310 (*p* < 0.05), as detailed in Table 1.

**Table 1:** Degree of coordination of expert opinions.

Indicator	First round			Second round		
	Kendall W	*χ2*	*P*	Kendall W	*χ2*	*P*
Primary indicator importance	0.275	8.811	0.012	0.310	9.909	0.007
Secondary indicator importance	0.263	63.127	< 0.001	0.300	67.245	< 0.001
Rationality of calculation formula	0.136	30.444	0.007	0.217	45.076	< 0.001
Applicability of data collection methods	0.149	35.642	0.002	0.259	58.063	< 0.001

### 5.3 Indicator modifications

Through a literature review and analysis, the research team identified 3 primary indicators and 18 secondary indicators. Following the semistructured expert interviews, 2 indicators (“composition ratio of hemodialysis nurses” and “arteriovenous fistula mapping rate”) were removed, while 2 indicators (“correct hand hygiene rate” and “hand hygiene compliance rate”) were merged into “correct execution of hand hygiene rate.” Additionally, 1 new indicator (“establishment of a vascular access monitoring team”) was added. A preliminary questionnaire comprising 3 primary indicators and 16 secondary indicators for hemodialysis vascular access nursing quality assessment was drafted and submitted for expert consultation.

In the first round, 11 experts proposed modifications to the secondary indicators. The research team adjusted the indicators on the basis of expert opinions and screening criteria. Two indicators with an average importance score ≤ 3.5 were removed: “ultrasound utilization rate for new fistula assessment” and “incidence of poor internal fistula function.” Two indicators were revised: “incidence of arteriovenous fistula thrombosis” was changed to “incidence of arteriovenous fistula aneurysms,” and “physical examination execution rate for arteriovenous fistula” was revised to “correct execution rate of physical examination for arteriovenous fistula. “One new indicator was added: “correct disinfection rate for catheter interface.”

**Figure 3:**
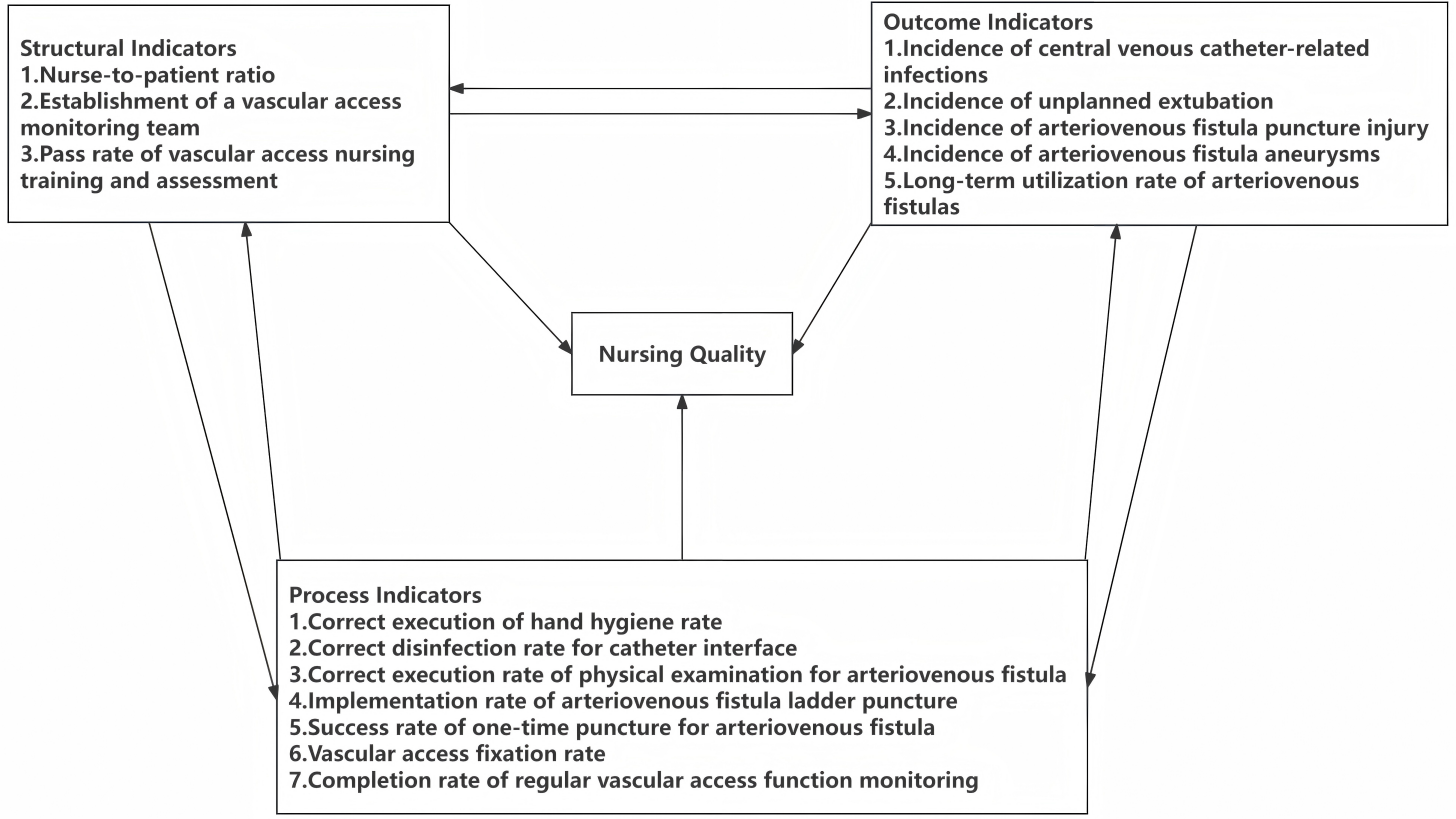
Logical framework of the nursing-sensitive quality indicator system for hemodialysis vascular access.

During the second round of correspondence, 16 experts re-evaluated the adjusted indicator system, proposing to modify the “incidence of arteriovenous fistula puncture bleeding/hematoma” to “incidence of arteriovenous fistula puncture injury.” The results revealed that the mean importance scores of all the indicators were >3.5, with a coefficient of variation < 0.25, indicating that a consensus was reached. On the basis of the results of the statistical analysis and clinical practice, the research team ultimately identified 3 primary indicators and 15 secondary indicators. The specific data and content are presented in Tables 2, 3. A logical framework diagram of the vascular access nursing quality-sensitive indicator system is presented in Figure 3.

**Table 2:** Results of expert consultation on nursing-sensitive quality indicators for hemodialysis vascular access.

Primary indicators	Secondary indicators	Importance			Rationality of calculation formula			Applicability of data collection methods		
		Scores (Mean ±SD)	CV	Percentage of full marks (%)	Scores (Mean ±SD)	CV	Percentage of full marks (%)	Scores (Mean ±SD)	CV	Percentage of full marks (%)
I Structural indicator		4.750 ± 0.447	0.094	75.00	/	/	/	/	/	/
	I-1 Nurse-to-patient ratio	4.938 ± 0.242	0.049	93.75	4.625 ± 0.696	0.150	75.00	4.313 ± 0.682	0.158	43.75
	I-2 Establishment of a vascular access monitoring team	4.813 ± 0.527	0.109	87.50	/	/	/	4.875 ± 0.331	0.068	87.50
	I-3 Pass rate of vascular access nursing training and assessment	4.563 ± 0.704	0.154	68.75	4.750 ± 0.433	0.091	75.00	4.625 ± 0.484	0.105	62.50
II Process indicator		4.313 ± 0.793	0.184	50.00	/	/	/	/	/	/
	II-1 Correct execution of hand hygiene rate	4.938 ± 0.242	0.049	93.75	4.938 ± 0.242	0.049	93.75	4.938 ± 0.242	0.049	93.75
	II-2 Correct disinfection rate for catheter interface	4.625 ± 0.484	0.105	62.50	4.813 ± 0.390	0.081	81.25	4.063 ± 0.899	0.221	43.75
	II-3 Correct execution rate of physical examination for arteriovenous fistula	4.875 ± 0.331	0.068	87.50	4.938 ± 0.242	0.049	93.75	4.938 ± 0.242	0.049	93.75
	II-4 Implementation rate of arteriovenous fistula ladder puncture	4.750 ± 0.433	0.091	75.00	4.625 ± 0.599	0.130	68.75	4.625 ± 0.484	0.105	62.50
	II-5 Success rate of one-time puncture for arteriovenous fistula	4.250 ± 0.661	0.156	37.50	4.313 ± 0.768	0.178	50.00	4.375 ± 0.781	0.178	56.25
	II-6 Vascular access fixation rate	4.438 ± 0.609	0.137	50.00	4.625 ± 0.484	0.105	62.50	4.750 ± 0.433	0.091	75.00
	II-7 Completion rate of regular vascular access function monitoring	4.000 ± 0.707	0.177	25.00	4.375 ± 0.696	0.159	50.00	4.625 ± 0.599	0.130	68.75
III Outcome indicator		4.688 ± 0.479	0.102	68.75	/	/	/	/	/	/
	III-1 Incidence of central venous catheter-related infections	5.000 ± 0.000	0	100	5.000 ± 0.000	0	100	4.938 ± 0.242	0.049	93.75
	III-2 Incidence of unplanned extubation	4.688 ± 0.464	0.099	68.75	4.813 ± 0.527	0.109	87.50	4.938 ± 0.242	0.049	93.75
	III-3 Incidence of arteriovenous fistula puncture injury	4.625 ± 0.599	0.130	68.75	4.500 ± 0.612	0.136	56.25	4.625 ± 0.599	0.130	68.75
	III-4 Incidence of arteriovenous fistula aneurysms	4.313 ± 0.768	0.178	50.00	4.438 ± 0.788	0.178	62.50	4.250 ± 0.829	0.195	50.00
	III-5 Long-term utilization rate of arteriovenous fistulas	4.938 ± 0.242	0.049	93.75	4.750 ± 0.433	0.091	75.00	4.688 ± 0.464	0.099	68.75

**Table 3:** Nursing quality-sensitive indicator system of hemodialysis vascular access.

Primary indicators	Secondary indicators	Definition	Calculation formula	Data collection method
I Structural indicator	I-1 Nurse-to-patient ratio	It refers to the ratio of the number of hemodialysis attending nurses to the number of hemodialysis patients under their care during the statistical period. This does not include the number of attending nurses and patients undergoing Continuous Renal Replacement Therapy (CRRT) (with a standard of 1:5 nurses to patients per shift).	1: sum of the number of hemodialysis patients during the same period/Total number of hemodialysis attending nurses during the statistical period	View the schedule and patient treatment count
	I-2 Establishment of a vascular access monitoring team	The vascular access monitoring team consists of nephrologists, dialysis nurses, vascular access physicians, radiologists, and dialysis access coordinators.	None	On-site observation
	I-3 Pass rate of vascular access nursing training and assessment	The ratio of the number of nurses who pass both the theoretical and practical assessments related to vascular access within the statistical period to the total number of specialized hemodialysis nurses.	Number of qualified nursing training assessments/Total number of hemodialysis specialists during the statistical period ^*^ 100%	Archival record collection method (assessment registration form)
II Process indicator	II-1 Correct execution of hand hygiene rate	The ratio of the number of correct hand hygiene practices performed by healthcare workers during a specific hand hygiene moment to the number of hand hygiene practices that should have been performed by healthcare workers during that hand hygiene moment within the statistical period. **Hand hygiene moments** refer to the five recommended hand hygiene moments outlined in the WHO Health Guidelines. **Correct hand hygiene** involves following the ‘seven-step' handwashing technique or the handwashing methods specified by the hospital.	Number of correct hand hygiene practices performed by healthcare workers during a specific hand hygiene moment/Number of hand hygiene practices that healthcare workers should perform during the statistical period at that hand hygiene moment^*^100%	On-site observation method, archival record collection method (daily supervision form)
	II-2 Correct disinfection rate for catheter interface	The ratio of the number of catheter interface disinfection cases performed correctly by nurses in accordance with national or hospital regulations during the statistical period to the total number of catheter interface disinfection cases.	Number of catheter interface disinfection cases correctly performed/Total number of catheter interface disinfection cases during the statistical period ^*^ 100%	On-site observation method, archival record collection method (daily supervision form)
	II-3 Correct execution rate of physical examination for arteriovenous fistula	The ratio of the number of patients who underwent correct physical examinations by nurses before arteriovenous fistula puncture during the statistical period to the total number of arteriovenous fistula puncture procedures.	Number of correct physical examinations performed before arteriovenous fistula puncture/Total number of arteriovenous fistula puncture cases within the statistical period ^*^ 100%	On-site observation method, archival record collection method (daily supervision form)
	II-4 Implementation rate of arteriovenous fistula ladder puncture	The ratio of the number of patients who actually underwent rope ladder puncture during the statistical period to the total number of patients who should have undergone this procedure.	Number of patients who actually underwent rope ladder puncture/Total number of patients who should have undergone this puncture during the statistical period ^*^ 100%	On-site observation method, archival record collection method (daily supervision form)
	II-5 Success rate of one-time puncture for arteriovenous fistula	The ratio of the number of successful one-time arteriovenous fistula punctures to the total number of arteriovenous fistula punctures during the statistical period	Number of successful one-time arteriovenous fistula punctures/Total number of arteriovenous fistula punctures within the statistical period ^*^ 100%	On-site observation method, archival record collection method (daily supervision form)
	II-6 Vascular access fixation rate	The ratio of the number of catheter/puncture needle secure fixation cases to the total number of dialysis patients during the statistical period	Number of stably secured vascular access cases/Total number of dialysis patients during the statistical period ^*^ 100%	On-site observation method, archival record collection method (daily supervision form)
	II-7 Completion rate of regular vascular access function monitoring	The ratio of the number of patients who completed regular monitoring of vascular access function during the statistical period to the total number of dialysis patients.	Number of patients with completed vascular access function monitoring/total number of dialysis patients during the statistical period ^*^ 100%	On-site observation method, archival record collection method (daily supervision form)
III Outcome indicator	III-1 Incidence of central venous catheter-related infections	The ratio of the number of central venous catheter-related infections to the total number of days the patient's central venous catheter was indwelled during the statistical period. The number of cases of exit infection, tunnel infection, and catheter-related bloodstream infection were counted separately.	Number of central venous catheter-related infections/Total days of central catheter placement during the statistical period ^*^ 1000‰	Archival record collection method (hemodialysis information system, outpatient dialysis event form)
	III-2 Incidence of unplanned extubation	It refers to the ratio of unplanned catheter removal cases to the total number of dialysis sessions during the statistical period. Unplanned hemodialysis catheter removal includes both unplanned catheter removal and unplanned removal of puncture needles.	Number of unplanned extubation cases/Total dialysis cases during the statistical period^*^100%	Archival record collection method (Adverse Event Reporting System)
	III-3 Incidence of arteriovenous fistula puncture injury	The ratio of the number of cases of arteriovenous fistula puncture injuries to the total number of hemodialysis cases via arteriovenous fistulas during the statistical period, including both autologous and graft arteriovenous fistulas.	Number of puncture injuries in arteriovenous fistulas/Total number of hemodialysis patients with arteriovenous fistulas during the statistical period ^*^ 1000‰	Archival record collection method (hemodialysis information system, daily supervision forms)
	III-4 Incidence of arteriovenous fistula aneurysms	The ratio of the number of arteriovenous fistulas (AVFs) with aneurysms to the total number of AVFs during the statistical period. In this definition, an aneurysm is defined as a vessel with an internal diameter exceeding three times that of the adjacent normal vessel and an internal diameter>2 cm.	Number of arteriovenous fistulas with aneurysms/Total number of arteriovenous fistulas during the statistical period ^*^ 100%	Archival record collection method (hemodialysis information system, daily supervision forms)
	III-5 Long-term utilization rate of arteriovenous fistulas	Proportion of maintenance hemodialysis patients with the same arteriovenous fistula in continuous use for>2 years during the statistical period	Number of patients with the same arteriovenous fistula undergoing maintenance hemodialysis for >2 years/Total number of maintenance hemodialysis patients during the statistical period ^*^ 100%	Archival record collection method (hemodialysis information system)

### 5.4 Weights of indicators

Among the three primary indicators, the structural indicator had the highest weight (0.6483), followed by the outcome indicator (0.2297), while the process indicator had the lowest weight (0.1220). Among the secondary indicators, the nurse-to-patient ratio ranked first (0.3498), followed by establishment of a vascular access monitoring team (0.1925), and pass rate of vascular access nursing training assessment (0.1059). The CRs for all pairwise comparison matrices were below 0.1 (structural indicators: CR = 0.0079; process indicators: CR = 0.0437; outcome indicators: CR = 0.0341), indicating acceptable consistency, as shown in Table 4.

**Table 4:** Relative importance of hemodialysis vascular access nursing quality-sensitive indicators.

Primary indicators	Weight	Secondary indicators	Weight	Combined weight(rank)	λmax	CR
I Structural indicator	0.6483	I-1 Nurse-to-patient ratio	0.5396	0.3498(1)	3.0092	0.0079
		I-2 Establishment of a vascular access monitoring team	0.2970	0.1925(2)		
		I-3 Pass rate of vascular access nursing training and assessment	0.1634	0.1059(3)		
II Process indicator	0.1220	II-1 Correct execution of hand hygiene rate	0.3231	0.0394(7)	7.3460	0.0437
		II-2 Correct disinfection rate for catheter interface	0.1467	0.0179(11)		
		II-3 Correct execution rate of physical examination for arteriovenous fistula	0.2543	0.0310(8)		
		II-4 Implementation rate of arteriovenous fistula ladder puncture	0.1492	0.0182(10)		
		II-5 Success rate of one-time puncture for arteriovenous fistula	0.0397	0.0048(14)		
		II-6 Vascular access fixation rate	0.0654	0.0080(13)		
		II-7 Completion rate of regular vascular access function monitoring	0.0216	0.0026(15)		
III Outcome indicator	0.2297	III-1 Incidence of central venous catheter-related infections	0.4569	0.1049(4)	5.1529	0.0341
		III-2 Incidence of unplanned extubation	0.1883	0.0433(6)		
		III-3 Incidence of arteriovenous fistula puncture injury	0.1073	0.0246(9)		
		III-4 Incidence of arteriovenous fistula aneurysms	0.0527	0.0121(12)		
		III-5 Long-term utilization rate of arteriovenous fistulas	0.1948	0.0447(5)		

## 6 Discussion

This study adopted the Donabedian “structure–process–result” three-dimensional quality framework as its theoretical basis. A literature review, semi-structured interviews, and Delphi expert surveys were used to construct a hemodialysis vascular access nursing quality indicator system. The integration of the analytic hierarchy process (AHP) reduced the subjective errors inherent in the Delphi method. The research methodology and procedures were rigorous, and the findings are grounded in the combined input of the expert panel. The experts invited to participate in this study possessed extensive theoretical knowledge and clinical practical experience, with all holding bachelor's degrees or higher and having at least 10 years of professional experience. They demonstrated both academic insights and clinical expertise in establishing sensitive indicator systems. The response rate for the two rounds of written questionnaires reached 100%, with experts proposing 23 revision suggestions regarding the indicator content, reflecting their high level of engagement in this research. The expert authority coefficient was 0.904, indicating the high credibility of the written consultation experts. The Kendall's W ranged from 0.136 to 0.310, and the coefficients of variation (CV) of the finally retained indicators were all < 0.25. Although the Kendall's W values were statistically significant (*p* < 0.05), the magnitude of these values (0.136–0.310) indicates moderate consistency.

In this study, structural indicators held the greatest weight, indicating that they serve as the foundation for ensuring the quality of vascular access care for hemodialysis patients. The allocation of resources, personnel qualifications, and institutional development at the structural level directly determines the standardization of the nursing process and the safety of outcomes. Under the current status of vascular access nursing management, the core differences in vascular access nursing quality first originate from disparities in resource allocation at the structural level: whether medical institutions are equipped with necessary human resources, have established dedicated vascular access nursing teams, and have developed standardized operating procedures are the core prerequisites determining the standardization of nursing processes and patient outcomes. In institutions where structure indicators do not meet the required standards, even if high requirements are set for process indicators, it is difficult to achieve continuous quality improvement. However, this does not mean that process and outcome indicators are unimportant: process indicators are the core of quality improvement, and outcome indicators are the direct reflection of quality level. The three dimensions of indicators support each other and jointly form a complete quality evaluation chain.

Structural indicators include the nurse-to-patient ratio, the establishment of a vascular access monitoring team, and the pass rate of vascular access nursing training and assessment. Among these, the nurse-to-patient ratio had the highest combined weight (0.3498), followed by the indicator of establishing a vascular access monitoring team, highlighting the importance of adequate nursing staffing in ensuring vascular access care quality. The nurse-to-patient ratio reflects the rationality of human resource allocation, and directly affects the timeliness and continuity of nursing practice. Particularly during high-load hemodialysis shifts, sufficient and stable nursing staffing serves as the first line of defense against complications including puncture injuries, hematomas, and catheter-related infections. Inadequate nurse staffing will directly lead to an excessive number of patients per nurse, which in turn reduces the standardization of vascular access assessment and maintenance procedures, and elevates the risk of adverse events such as unplanned extubation and catheter-related infections. These findings are consistent with the study by Balouchi et al. (25). Additionally, studies by McIntyre et al. (20) in Canada and Gao et al. (21) in China have also verified the negative correlation between the nurse-to-patient ratio and the incidence of adverse events in hemodialysis patients. In California, the United States, the required nurse-to-patient ratio for hemodialysis settings is 1:8 (26); in South Korea it is 1:5 to 1:6 (27); in Australia it ranges from 1:3 to 1:4 (28); and in China it is 1:5 (29). Studies have shown that the ratio of hemodialysis nurses to patients affects patient prognosis, and a smaller number of nurses directly affects treatment adherence and safety, resulting in increased rates of missed diagnoses, hospitalization, and mortality (30). Currently, only 34.50% of hemodialysis centers in tertiary hospitals in China meet the aforementioned nurse-to-patient ratio requirement (31), indicating a widespread gap in the allocation of hemodialysis nursing human resources nationwide. There is an urgent need for relevant authorities to issue targeted policies to promote the scientific and rational allocation of nursing manpower. In clinical management practice, the nurse-to-patient ratio can be incorporated into the regular assessment system for hemodialysis centers, which will incentivize managers to prioritize investment in nursing staffing, improve the construction of specialized vascular access nursing teams, and further facilitate the transformation of hemodialysis centers from the traditional extensive management model of “valuing treatment over nursing” to a refined management model centered on nursing quality.

The establishment of a vascular access monitoring team is an effective measure to reduce vascular access-related complications. The team is mainly composed of specialized vascular access nurses, whose core responsibilities include vascular access assessment, operation quality supervision, early identification of complications, and specialized training for nursing staff (32). As the long-term outcomes of patients' vascular access are directly related to the quality of such nursing work, including “the establishment of a vascular access monitoring team” as a structural indicator has clear clinical significance: it can promote the construction of a stable specialized nursing talent team in hemodialysis centers, reduce nursing quality fluctuations caused by staff turnover, and ensure patient safety from the source of management. However, the implementation of structural indicators still faces practical obstacles. First, strict implementation of the nurse-to-patient ratio standard will bring considerable short-term labor cost pressure to hemodialysis centers with a large patient base and insufficient human resource reserves. Second, specialized vascular access training and routine operation of the monitoring team require sustained financial support, and many for-profit dialysis institutions have low willingness to invest in such work due to cost control concerns. These limitations need to be gradually addressed by administrative departments through layered assessment and supporting policies, so as to give full play to the guiding role of structure indicators in nursing management. However, the determination of indicator thresholds needs to be adaptively adjusted in alignment with the medical resource allocation level, medical insurance policies, and cultural characteristics of different countries and regions.

The process indicators include seven secondary indicators, among which the correct execution of hand hygiene rate has the highest combined weight (0.0394), followed by the correct execution rate of physical examination for arteriovenous fistula (0.0310). The correct implementation of hand hygiene refers to performing hand hygiene with the correct method at the right time. Hemodialysis patients are at high risk of hospital-acquired infections, and the correct implementation of hand hygiene is closely related to the occurrence of patient infections. In other countries, the correct hand hygiene implementation rate is also a key monitoring indicator for hemodialysis centers. A study conducted in Mexico confirmed that targeted hand hygiene monitoring can effectively improve hand hygiene compliance, which in turn improves the performance of a series of infection control and care quality indicators (33). China's “Standard Operating Procedures for Blood Purification (2021 Edition)” (34) explicitly requires hand hygiene as a mandatory core step before and after vascular access procedures, but the actual implementation rate remains low. A Chinese multi-center study (35) revealed that the compliance rate of nurses with hand hygiene timing and methods during hemodialysis pre-wash operations was low (76.62%), with major issues including nurses lacking hand hygiene awareness after completing equipment and consumable checks, using hand sanitizer to rub hands instead of washing them before entering the treatment environment, incorrect hand sanitizer squeezing techniques, and some replacing hand washing with wearing gloves. A study by Moon and Jang (36) in South Korea also found that only 33% of medical staff perform standardized hand hygiene after removing gloves, indicating that insufficient hand hygiene compliance is a common problem at home and abroad. It is urgent to intervene by strengthening standardized training and improving full-process quality control to reduce the risk of vascular access-related infections. From a public health perspective, hand hygiene is the most cost-effective measure for infection prevention and control. On one hand, it can directly reduce the incidence of vascular access complications such as catheter-related bloodstream infections, alleviate patients' hospitalization burden, lower mortality risk, reduce the cross-transmission of drug-resistant bacteria, and save public medical resources. On the other hand, as a basic quality control indicator with high homogeneity and low implementation difficulty, hand hygiene can rapidly narrow the nursing quality gap between hemodialysis centers of different levels, and comprehensively improve the safety guarantee level of hemodialysis patients.

Physical examination of arteriovenous fistula refers to a monitoring method for identifying dysfunctional arteriovenous fistulas by assessing stenosis through visual inspection, palpation, and auscultation combined with the arm-raising and pulsation enhancement tests. This approach offers advantages such as simplicity, time efficiency, and relatively accurate evaluation of fistula maturity and complications. Caputo et al. (37) reported that when ultrasound Doppler was used as the gold standard, physical examination showed a sensitivity of 53% for detecting early functional loss of internal fistulas, while the specificity for identifying normal arteriovenous fistulas (AVFs) reached 98%. Rodrigues et al. (38) reported that physical examination revealed that experienced nurses achieved greater accuracy (80%) in predicting AVF maturity. A British guideline recommended performing a physical examination before each arteriovenous fistula puncture to ensure that blood flow meets dialysis prescription requirements (39). However, the actual execution rate of physical examination for arteriovenous fistula by hemodialysis nurses remains low in clinical practice. Liu et al. (40) reported that only 40.65% of hemodialysis units in Beijing conducted comprehensive physical examinations prior to internal fistula punctures, with 48.72% in tertiary hospitals and less than 30% in nontertiary hospitals. All these rates are far lower than the 50%−96% implementation rate reported in a series of international studies (41–43). The reasons for this are largely attributed to nurses' insufficient recognition of the clinical value of physical examinations, the lack of standardized operational procedures, and the absence of assessment mechanisms. Therefore, incorporating this indicator into the vascular access nursing quality monitoring system can help increase hemodialysis nurses‘ awareness of the importance of physical examinations and promote the establishment of training systems and standardized operational checklists, thereby effectively reducing the failure rate of internal fistulas and ensuring adequate dialysis and long-term vascular access safety for patients. However, the core barrier to the implementation of such process indicators lies in the poor feasibility of continuous data collection. Data such as hand hygiene and physical assessment implementation rates need to be collected through real-time observation, which requires additional manpower and time investment from hemodialysis centers. However, clinical nurses are generally saturated with heavy workload, making it difficult to undertake extra monitoring tasks. It is recommended to adopt the mode of “sampling inspection and key monitoring” for process indicator monitoring, and qualified hemodialysis centers can also explore the use of artificial intelligence tools to complete automatic data collection and management.

The outcome indicators included five secondary metrics, with the incidence of central venous catheter-related infections (CVCIs) (0.1049) having the highest combined weight, followed by the long-term utilization rate of arteriovenous fistulas (0.0447). CVCIs have become the second leading cause of mortality among hemodialysis patients (44). Hemodialysis-related CVCIs primarily include catheter exit site infections, catheter tunnel infections, and catheter-related bloodstream infections (CRBSI). Notably, the incidence of CRBSI has long been a core indicator highlighted in international clinical guidelines (45, 46). Data from the National Health care Security Network (NHSN) (47) indicate a 2.16% CVCIs rate, with 70% of cases occurring in long-term catheterization patients. However, surveillance data on exit site infections and tunnel infections remain scarce in the literature. This study is the first to propose separate monitoring of the three indicators, which facilitates early clinical detection of catheter infection signs and advances the prevention of infection, thereby avoiding severe CRBSI in patients. This has significant clinical implications for improving the quality of vascular access care. The long-term utilization rate of arteriovenous fistulas directly reflects the maintenance capacity of vascular access and the efficacy of nursing interventions. The stable long-term use of arteriovenous fistulas can reduce the dependence on central venous catheters, as well as the risks of complications such as infections and thrombosis, thereby decreasing hospitalization rates and medical burdens. This, in turn, enhances dialysis adequacy and quality of life, making it a critical outcome of vascular access that is of shared concern to health care providers and patients. Monitoring this indicator helps identify issues in arteriovenous fistula maintenance, puncture techniques, and complication prevention, providing data support for optimizing nursing workflows. The application of outcome indicators enables managers to accurately identify weak links in nursing quality and verify the effectiveness of quality improvement interventions. For instance, if a hemodialysis center detects a higher-than-national-average incidence of catheter-related infections through routine monitoring, it can trace back to structural and process indicators (e.g., the correct execution of hand hygiene rate, nurse-to-patient ratio) to locate the root cause, develop targeted improvement strategies, and validate the improvement effect via subsequent outcome indicator data, thus forming a complete closed-loop management workflow. In addition, outcome indicators can provide evidence support for policy formulation. Currently, China lacks a national vascular access nursing quality data collection platform, making it difficult to accurately identify quality gaps across different regions. If a national or provincial dialysis quality monitoring database is established based on the outcome indicators proposed in this study, core data such as national-level catheter infection incidence and long-term arteriovenous fistula (AVF) utilization rate can be obtained, which will provide data support for health administrative departments to adjust policies on manpower allocation, medical insurance reimbursement, and primary care capacity building. Since this study did not define predefined thresholds for the outcome indicators, other countries or regions can make adaptive adjustments to the indicator thresholds according to their local healthcare contexts and resource availability in subsequent clinical applications.

### 6.1 Limitations

This study has several limitations. First, the indicator system developed in this work has only been validated via expert consensus to date. Owing to constraints in human resources, material support, and research timelines, it has not yet been tested in real clinical settings. Its practical operability, data accessibility, inter-rater reliability, as well as associations with clinical outcomes including vascular access-related adverse event rates and patient satisfaction, remain to be verified in further empirical studies. For subsequent research, a single-center pre-experiment can be first conducted to preliminarily evaluate the operability and clinical adaptability of the indicators. Following optimization and adjustment, a multi-center, large-sample clinical validation study should be carried out to further clarify the clinical application value of this indicator system. In addition, longitudinal follow-up studies can be performed to dynamically observe the long-term change trends of the indicators, so as to provide reference for continuous quality improvement. Second, all experts were recruited from within China, which may introduce regional and cultural biases. The panel also excluded patients and caregivers, although this was a deliberate choice given the study's aim of constructing a clinically grounded nursing quality measurement tool, for which frontline nurses, physicians, and nursing managers are the primary knowledge holders. Future validation studies should consider incorporating these broader perspectives. Additionally, application of this system outside China will require adaptive adjustments and local validation according to regional healthcare contexts.

## 7 Conclusion

This study is based on Donabedian's three-dimensional quality evaluation model and it systematically constructed a sensitive indicator system for evaluating the quality of hemodialysis vascular access nursing through a combination of methods, including literature review, semistructured interviews, the Delphi method, and an analytic hierarchy process, with weight assignment completed. During the research process, two rounds of expert consultation and validation were conducted, ultimately forming an indicator framework comprising 3 primary indicators and 15 secondary indicators. This system combines standardization with a systematic methodological approach, aligns with clinical nursing practice scenarios and nursing management needs, and provides a reference for nursing managers to implement precision nursing quality control.

## Data Availability

The datasets presented in this article are not readily available because the datasets generated and analyzed during the current study are not publicly available due to the sensitivity of expert opinions and ethical restrictions, but are available from the corresponding author on reasonable request. Requests to access the datasets should be directed to clhxxuetou@163.com.
