# Supplementary material for: Construction of nursing-sensitive quality indicators for hemodialysis vascular access: a Delphi study
Source: Front Public Health. 2026 Jul 13;14:1858484. doi: 10.3389/fpubh.2026.1858484 (PMC13422778; doi:10.3389/fpubh.2026.1858484)
Supplement: Supplementary file 2 [file Table_2.docx]

Supplementary Material Table 2 Full Search Strategies for Each Database

**Appendix. The search strategies**

| **Databases** | **Step** | **Search Strategies** | **Results** |
| --- | --- | --- | --- |
| PubMed | #1 | "renal dialysis"[MeSH Terms] OR "dialys* renal"[Title/Abstract] OR "renal dialys*"[Title/Abstract] OR "hemodialys*"[Title/Abstract] OR "dialys* extracorporeal"[Title/Abstract] OR "extracorporeal dialys*"[Title/Abstract] OR "hemodiafiltration"[MeSH Terms] OR "hemodialysis"[Title/Abstract] OR "MHD"[Title/Abstract] Sort by: Most Recent | 161,842 |
|  | #2 | "arteriovenous fistula"[MeSH Terms] OR "fistula* arteriovenous"[Title/Abstract] OR "arteriovenous fistula*"[Title/Abstract] OR "arteriovenous aneurysm"[Title/Abstract] OR "aneurysm arteriovenous"[Title/Abstract] OR "central venous catheters"[MeSH Terms] OR "catheter* central venous"[Title/Abstract] OR "venous catheter* central"[Title/Abstract] OR "central venous catheter"[Title/Abstract] OR "vascular access"[Title/Abstract] Sort by: Most Recent | 53,233 |
|  | #3 | "quality indicators, health care"[MeSH Terms] OR "quality indicator* healthcare"[Title/Abstract] OR "healthcare quality indicator*"[Title/Abstract] OR "indicator* healthcare quality"[Title/Abstract] OR "health metric*"[Title/Abstract] OR "metrics health"[Title/Abstract] OR ((("Global"[All Fields] OR "globalism"[All Fields] OR "globalize"[All Fields] OR "globalized"[All Fields] OR "globalizes"[All Fields] OR "globalizing"[All Fields] OR "globally"[All Fields] OR "globals"[All Fields] OR "internationality"[MeSH Terms] OR "internationality"[All Fields] OR "globalization"[All Fields]) AND ("precipitating factors"[MeSH Terms] OR ("precipitating"[All Fields] AND "factors"[All Fields]) OR "precipitating factors"[All Fields] OR "Trigger"[All Fields] OR "triggers"[All Fields] OR "triggerable"[All Fields] OR "triggered"[All Fields] OR "triggering"[All Fields] OR "triggerings"[All Fields]) AND "Tool"[All Fields]) AND "Healthcare"[Title/Abstract]) OR "healthcare global trigger tool"[Title/Abstract] OR "nursing quality"[Title/Abstract] OR "sensitive indicator"[Title/Abstract] OR "quality assessment*"[Title/Abstract] OR "quality indicator*"[Title/Abstract] OR "quality improvement"[MeSH Terms] OR "improvement* quality"[Title/Abstract] OR "quality improvements"[Title/Abstract] Sort by: Most Recent | 124,496 |
|  | #4 | #1 AND #2 AND #3 | 143 |
| Web of Science | #1 | **TS=(renal dialysis OR Dialys*, Renal OR Renal Dialys* OR Hemodialys* OR Dialys*, Extracorporeal OR Extracorporeal Dialys* OR Hemodiafiltration OR hemodialysis OR MHD)** | 211,485 |
|  | #2 | **TS=(Arteriovenous Fistula OR Fistula*, Arteriovenous OR Arteriovenous Fistula* OR Arteriovenous Aneurysm OR Aneurysm, Arteriovenous OR Central Venous Catheters OR Catheter*, Central Venous OR Venous Catheter*, Central OR Central Venous Catheter OR vascular access)** | 70,987 |
|  | #3 | TS=(Quality Indicators, Health Care OR Quality Indicator*, Healthcare OR Healthcare Quality Indicator* OR Indicator*, Healthcare Quality OR Health Metric* OR Metrics, Health OR Global Trigger Tool, Healthcare OR Healthcare Global Trigger Tool OR nursing quality OR sensitive indicator OR quality assessment* OR quality indicator* OR Quality Improvement OR Improvement*, Quality OR Quality Improvements) | 1,108,745 |
|  | #4 | #1 AND #2 AND #3 | 556 |
| EMBASE | **#1** | **(renal dialysis or Dialys*, Renal or Renal Dialys* or Hemodialys* or Dialys*, Extracorporeal or Extracorporeal Dialys* or Hemodiafiltration or hemodialysis).ab.** | 119,679 |
|  | **#2** | **(Arteriovenous Fistula or Fistula*, Arteriovenous or Arteriovenous Fistula* or Arteriovenous Aneurysm or Aneurysm, Arteriovenous or Central Venous Catheters or Catheter*, Central Venous or Venous Catheter*, Central or Central Venous Catheter or vascular access).ab.** | 58,695 |
|  | **#3** | **(Quality Indicators, Health Care or Quality Indicator*, Healthcare or Healthcare Quality Indicator* or Indicator*, Healthcare Quality or Health Metric* or Metrics, Health or Global Trigger Tool, Healthcare or Healthcare Global Trigger Tool or nursing quality or sensitive indicator or quality assessment* or quality indicator* or Quality Improvement or Improvement*, Quality or Quality Improvements).ab.** | 163,285 |
|  | #4 | #1 AND #2 AND #3 | 182 |
| Cochrane Library | #1 | MeSH descriptor: [Renal Dialysis] explode all trees | 7,566 |
|  | #2 | MeSH descriptor: [Hemodiafiltration] explode all trees | 384 |
|  | #3 | (hemodialysis):ti,ab,kw | 16,162 |
|  | #4 | #1 OR #2 OR #3 | 18,146 |
|  | #5 | MeSH descriptor: [Arteriovenous Fistula] explode all trees | 248 |
|  | #6 | MeSH descriptor: [Central Venous Catheters] explode all trees | 329 |
|  | #7 | (vascular access):ti,ab,kw | 3,979 |
|  | #8 | #5 OR #6 OR #7 | 4,406 |
|  | #9 | MeSH descriptor: [Quality Indicators, Health Care] explode all trees | 1,357 |
|  | #10 | (nursing quality):ti,ab,kw | 10,815 |
|  | #11 | (sensitive indicator*):ti,ab,kw | 1,000 |
|  | #12 | (quality assessment*):ti,ab,kw | 89,267 |
|  | #13 | (quality indicator*):ti,ab,kw | 7,569 |
|  | #14 | MeSH descriptor: [Quality Improvement] explode all trees | 1,299 |
|  | #15 | #9 OR #10 OR #11 OR #12 OR #13 OR #14 | 103,184 |
|  | #16 | #4 AND #8 AND #15 | 82 |
| CINAHL | S1 | AB renal dialysis OR AB Dialys*, Renal OR AB Renal Dialys* OR AB Hemodialys* OR AB Dialys*, Extracorporeal OR AB Extracorporeal Dialys* OR AB Hemodiafiltration OR AB hemodialysis OR AB MHD | 139,893 |
|  | S2 | AB Arteriovenous Fistula OR AB Fistula*, Arteriovenous OR AB Arteriovenous Fistula* OR AB Arteriovenous Aneurysm OR AB Aneurysm, Arteriovenous OR AB Central Venous Catheters OR AB Catheter*, Central Venous OR AB Venous Catheter*, Central OR AB Central Venous Catheter OR AB vascular access | 57,465 |
|  | S3 | AB Quality Indicators, Health Care OR AB Quality Indicator*, Healthcare OR AB Healthcare Quality Indicator* OR AB Indicator*, Healthcare Quality OR AB Health Metric* OR AB Metrics, Health OR AB Global Trigger Tool, Healthcare OR AB Healthcare Global Trigger Tool OR AB nursing quality OR AB sensitive indicator OR AB quality assessment* OR AB quality indicator* OR AB Quality Improvement OR AB Improvement*, Quality OR AB Quality Improvements | 455,247 |
|  | S4 | S1 AND S2 AND S3 | 164 |
| CNKI |  | (TKA="透析") AND (TKA="动静脉内瘘" + "内瘘" + "导管" + "cuff导管" + "隧道式导管" + "通路") AND (TKA="质量评价" + "护理质量" + "敏感指标" + "质量改进" + "质量指标") | 557 |
| WanFang |  | 题名或关键词:(透析) and 题名或关键词:(动静脉内瘘 OR 内瘘 OR 导管 OR cuff导管 OR 隧道式导管 OR 通路) and 题名或关键词:(质量评价 OR 护理质量 OR 敏感指标 OR 质量改进 OR 质量指标) | 371 |
| CMGN |  | Search by "血液透析 质量" | 341 |
