# Supplementary material for: Construction of nursing-sensitive quality indicators for hemodialysis vascular access: a Delphi study
Source: Front Public Health. 2026 Jul 13;14:1858484. doi: 10.3389/fpubh.2026.1858484 (PMC13422778; doi:10.3389/fpubh.2026.1858484)
Supplement: Supplementary file 3 [file Table_3.docx]

Supplementary Material Table 3 Summary of included studies

| Title | Tool | Evaluation Outcome |
| --- | --- | --- |
| 持续质量改进对血液透析留置导管患者护理质量的影响^[1]^ | Johns Hopkins Nursing Evidence-based Practice (JHNEBP） | ⅡB |
| 持续质量改进联合护理质量指标在减少动静脉内瘘穿刺损伤中的应用研究^[2]^ |  | ⅡB |
| 持续质量改进在预防血液透析导管相关感染中的应用^[3]^ |  | ⅡB |
| 护理质量敏感指标在血液透析患者动静脉内瘘管理中的应用^[4]^ |  | ⅠB |
| 基于SHEL分析法的综合管理模式在提高MHD患者血管通路护理质量中的应用^[5]^ |  | ⅡB |
| 集束化护理在血透室护理敏感指标控制中的应用^[6]^ |  | ⅠB |
| 全程跟踪护理在维持性血液透析患者血管通路持续质量改进中的应用价值^[7]^ |  | ⅠB |
| 血液净化中心专科护理监测指标的制定与应用^[8]^ |  | ⅢB |
| 应用护理敏感质量指标提升血液透析质量^[9]^ |  | ⅢA |
| 应用血管通路记录表实施内瘘维护持续质量改进的研究^[10]^ |  | ⅡB |
| 自体动静脉内瘘功能不良监测相关指南的质量评价及内容分析^[11]^ |  | ⅣA |
| Donabedian质量理论在血液透析患者血管通路持续质量改进中的应用^[12]^ |  | ⅡB |
| PDCA循环管理在防治血液透析患者人工血管内瘘并发症中的应用^[13]^ |  | ⅠB |
| UK Kidney Association Clinical Practice Guideline on vascular access for haemodialysis.^[14]^ |  | ⅣA |
| Quality improvement in vascular access: The role of patient-reported outcome measures.^[15]^ |  | ⅤB |
| Monitoring of hemodialysis quality-of-care indicators: why is it important?^[16]^ |  | ⅢB |
| KDOQI Clinical Practice Guideline for Vascular Access: 2019 Update^[17]^ |  | ⅣA |
| Analysis of primary survival and quality indicators of native arteriovenous fistulas performed during the period 2020-2023^[18]^ |  | ⅢB |
| Quality Metrics in Dialysis: A Welcome Change for Vascular Access.^[19]^ |  | ⅤB |
| Adding New Components to a Composite Quality Metric: How Good Is Good Enough?^[20]^ |  | ⅢA |
| Effects of a Quality Improvement Program to Reduce Central Venous Catheter-Related Infections in Hemodialysis Patients.^[21]^ |  | ⅠB |
| Systematic review of risk prediction models for arteriovenous fistula dysfunction in maintenance hemodialysis patients^[22]^ |  | ⅠA |

References

[1] 陈文璇, 何筱娴, 李雪梅, 等. 持续质量改进对血液透析留置导管患者护理质量的影响[J]. 齐鲁护理杂志, 2013, 19(09): 71-72.

[2] 王颖, 梁俊卿, 鲁姣健, 等. 持续质量改进联合护理质量指标在减少动静脉内瘘穿刺损伤中的应用研究[J]. 护士进修杂志, 2024, 39(02): 164-168.

[3] 卢亚飞, 姚丽伟, 许敏霞, 等. 持续质量改进在预防血液透析导管相关感染中的应用[J]. 中华临床感染病杂志, 2021, 14(1): 70-74.

[4] 曹丽, 袁亚萍, 朱琳. 护理质量敏感指标在血液透析患者动静脉内瘘管理中的应用[J]. 国际护理学杂志, 2023, 42(20): 3683-3688.

[5] 鲁绍娟, 李玲, 欧喜金. 基于SHEL分析法的综合管理模式在提高MHD患者血管通路护理质量中的应用[J]. 齐鲁护理杂志, 2023, 29(07): 1-4.

[6] 陈付梅. 集束化护理在血透室护理敏感指标控制中的应用[J]. 实用临床护理学电子杂志, 2020, 5(51): 104.

[7] 骆美良, 骆瑾瑜, 沈洁, 等. 全程跟踪护理在维持性血液透析患者血管通路持续质量改进中的应用价值[J]. 护士进修杂志, 2019, 34(09): 774-776+780.

[8] 姜颖洁. 血液净化中心专科护理监测指标的制定与应用[J]. 中国乡村医药, 2020, 27(05): 78-79.

[9] 高菊林, 辛霞, 车文芳, 等. 应用护理敏感质量指标提升血液透析质量[J]. 中国卫生质量管理, 2018, 25(05): 67-70.

[10] 孙慧娟, 吴捷, 王春玲, 等. 应用血管通路记录表实施内瘘维护持续质量改进的研究[J]. 护理管理杂志, 2015, 15(06): 417-419.

[11] 刘瑶, 丁炎明, 李晶, 等. 自体动静脉内瘘功能不良监测相关指南的质量评价及内容分析[J]. 中华现代护理杂志, 2019, 25(36): 4719-4723.

[12] 陶珍晖, 郭学, 宋妍, 等. Donabedian质量理论在血液透析患者血管通路持续质量改进中的应用[J]. 中国血液净化, 2018, 17(02): 102-106.

[13] 孟秀云, 姜立萍, 陈荣, 等. PDCA循环管理在防治血液透析患者人工血管内瘘并发症中的应用[J]. 中国血液净化, 2009, 8(11): 635-636.

[14] Aitken E, Anijeet H, Ashby D, et al. UK Kidney Association Clinical Practice Guideline on vascular access for haemodialysis.[J]. BMC nephrology, 2025, 26(1): 461.

[15] Field M, Tullett K, Khawaja A, et al. Quality improvement in vascular access: The role of patient-reported outcome measures.[J]. The journal of vascular access, 2020, 21(1): 19-25.

[16] Grangé S, Hanoy M, Le Roy F, et al. Monitoring of hemodialysis quality-of-care indicators: why is it important?[J]. BMC nephrology, 2013, 14(1): 109.

[17] Lok C E, Huber T S, Lee T, et al. KDOQI Clinical Practice Guideline for Vascular Access: 2019 Update[J]. American Journal of Kidney Diseases, 2020, 75(4): S1-S164.

[18] Parra S.C.A., Murillo J.M., Calvo V.T., et al. Analysis of primary survival and quality indicators of native arteriovenous fistulas performed during the period 2020-2023[J]. Nephrology Dialysis Transplantation, 2025, 40(Supplement 3): i3030-i3031.

[19] Pujari A, Yuo T H, Lee T. Quality Metrics in Dialysis: A Welcome Change for Vascular Access.[J]. Advances in kidney disease and health, 2025, 32(5): 437-441.

[20] Salerno S, Yang E, Dahlerus C, et al. Adding New Components to a Composite Quality Metric: How Good Is Good Enough?[J]. Medical care, 2025, 63(4): 293-299.

[21] Yang Z, Ma X, Chen Y, et al. Effects of a Quality Improvement Program to Reduce Central Venous Catheter-Related Infections in Hemodialysis Patients.[J]. The American journal of the medical sciences, 2021, 361(4): 461-468.

[22] Yao S, Ma G, Dong Y, et al. Systematic review of risk prediction models for arteriovenous fistula dysfunction in maintenance hemodialysis patients[J]. PLOS ONE, 2025, 20(5): 1-16.
