# Supplementary material for: Construction of nursing-sensitive quality indicators for hemodialysis vascular access: a Delphi study
Source: Front Public Health. 2026 Jul 13;14:1858484. doi: 10.3389/fpubh.2026.1858484 (PMC13422778; doi:10.3389/fpubh.2026.1858484)
Supplement: Supplementary file 4 [file Table_4.docx]

**Supplementary Material Table 4 Characteristics of the expert panel**

| **ID** | **Gender** | **Education** | **Role** | **Professional title** | **Institution Type** | **Province** | **Years of vascular access experience** | **Delphi experience** |
| --- | --- | --- | --- | --- | --- | --- | --- | --- |
| 1 | Female | Bachelor | Registered nurse/Infection control nurse | Intermediate | Tertiary hospital | Yunnan | 30 | Yes |
| 2 | Female | Bachelor | Registered nurse | Intermediate | Tertiary hospital | Sichuan | 17 | Yes |
| 3 | Female | Bachelor | Registered nurse/Infection control nurse | Intermediate | Tertiary hospital | Sichuan | 19 | Yes |
| 4 | Female | Bachelor | Head nurse | Intermediate | Tertiary hospital | Beijing | 13 | Yes |
| 5 | Female | Bachelor | Head nurse | Senior | Tertiary hospital | Shanghai | 19 | Yes |
| 6 | Male | Master | Team leader (physician) | Senior | Tertiary hospital | Chongqing | 14 | Yes |
| 7 | Male | Bachelor | Head nurse | Intermediate | Tertiary hospital | Sichuan | 15 | Yes |
| 8 | Male | Bachelor | Registered nurse | Intermediate | Tertiary hospital | Chongqing | 14 | Yes |
| 9 | Male | Master | Head nurse | Intermediate | Tertiary hospital | Guangdong | 13 | Yes |
| 10 | Female | Bachelor | Head nurse | Senior | Tertiary hospital | Guangdong | 34 | Yes |
| 11 | Male | Master | Head nurse | Intermediate | Tertiary hospital | Hubei | 16 | Yes |
| 12 | Female | Bachelor | Head nurse | Senior | Tertiary hospital | Hubei | 30 | Yes |
| 13 | Female | Bachelor | Head nurse | Senior | Tertiary hospital | Sichuan | 28 | Yes |
| 14 | Female | Master | Team leader (physician) | Senior | Tertiary hospital | Shanghai | 18 | Yes |
| 15 | Female | Bachelor | Head nurse | Senior | Tertiary hospital | Shandong | 32 | Yes |
| 16 | Female | Master | Head nurse | Senior | Tertiary hospital | Fujian | 30 | Yes |
